# Nano-Elicitation Approaches for Boosting Secondary Metabolites in Medicinal Plant Cell Cultures

**DOI:** 10.3390/plants15010046

**Published:** 2025-12-23

**Authors:** Pooran Golkar, Edgar Vázquez-Núñez, José R. Peralta-Videa

**Affiliations:** 1 Department of Natural Resources, Isfahan University of Technology, Isfahan 84156-83111, Iran; poorangolkar@gmail.com; 2 Nano- and Biotechnological Applications for Sustainability Research Group (NanoBioTS), Division of Sciences and Engineering, Department of Chemical, Electronic and Biomedical Engineering, Laboratory for Environmental and Energy Sustainability (LaSAE), University of Guanajuato, Leon 37150, Guanajuato, Mexico; ed.vazquezn@gmail.com; 3 Department of Chemistry and Biochemistry, The University of Texas at El Paso, El Paso, TX 79968, USA

**Keywords:** cell culture, callus, hairy roots, nanomaterials, elicitor, metabolites

## Abstract

Medicinal plants are a rich source of diverse secondary metabolites (SMs) with significant industrial and medicinal applications. However, the natural content of these compounds is often low and influenced by various environmental and biological factors, making large-scale extraction from conventionally cultivated plants challenging. This review comprehensively examines the efficacy and benefits of plant in vitro culture techniques, specifically, callus, cell suspension, and hairy root cultures, for enhanced SMs production. A primary focus is placed on the elicitation effects of various nanomaterials and their mechanisms of action in boosting SMs synthesis. We present successful case studies utilizing different classes of nanomaterials, including metal oxides, non-metal oxides, carbon-based materials, polysaccharides, and quantum dots, as nano-elicitors. Furthermore, the review discusses the advantages and current challenges of nanomaterial-based elicitation, as well as its future applications and prospects. The insights consolidated in this review underscore the potential of nanoparticle-mediated elicitation as a robust strategy for the efficient production of valuable SMs in plant cell cultures. Finally, we emphasize the broad utility of diverse nanomaterials and highlight critical areas requiring further investigation in this field.

## 1. Introduction

Plants are a source of bioactive metabolites that are used in various fields, including food, flavors, agrochemicals, and therapeutic proteins [1]. Plant metabolites, divided into primary and secondary, play crucial roles in the growth and survival of plant species [1,2]. Primary metabolites (such as amino acids, carbohydrates, lipids, and proteins) directly support essential cellular processes, including respiration, photosynthesis, growth, and development [2]. Secondary metabolites (SMs) or specialized metabolites are produced from primary metabolites through different pathways [3].

Plant SMs have various functions, including signaling, stimulatory and inhibitory effects on enzymes, catalytic and defensive activities, and interactions with other organisms [1,2]. These metabolites also have different functions in plant growth, such as adjusting to environmental stresses (e.g., elevated O_3,_ ionizing radiation, and wounding) and shielding plants from pathogen infections [3,4]. The production of SMs is internally mediated by plant phytohormones, such as jasmonate, salicylic acid, and their derivatives, as signal transducers [3,5].

SMs are derived from primary metabolites by biosynthetic modifications, such as hydroxylation, glycosylation, and methylation [6]. SMs are considered key elements for ecosystem engagement [7,8]. Plant SMs have key applications across various industries, including pharmaceuticals, agriculture, cosmetics, dietary supplements, textiles, fragrances, flavors, dyes, and biostimulants [9].

**Figure 1:**
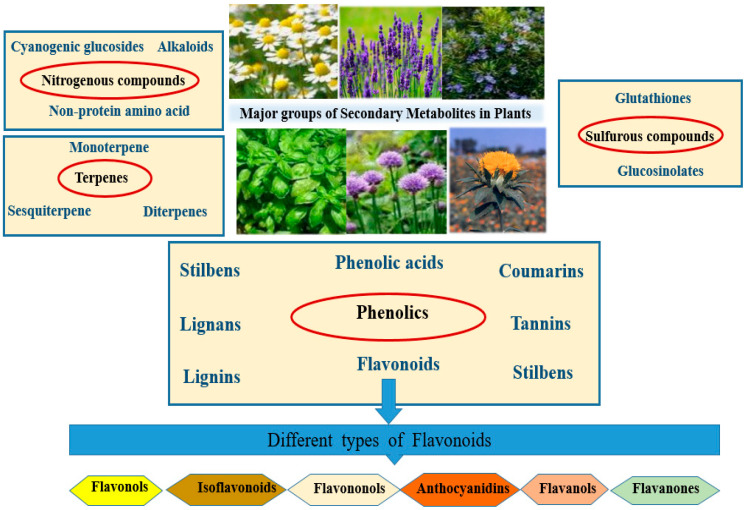
The four major classes of plant secondary metabolites in medicinal plants are sulfur compounds, nitrogenous compounds, terpenes, and phenolic compounds.

Plant SMs are classified based on their chemical structures, functional groups, and methods of synthesis [1]. The four main categories are as follows: **1**—Nitrogen-containing compounds, with main subgroups of alkaloids (e.g., morphine, quinine, nicotine, caffeine), cyanogenic glucosides (e.g., Dhurrin), and non-protein amino acids (e.g., azetidine). **2**—Terpenes, which are derived from isoprene (C_5_) units and comprise several subgroups with distinct compounds within each group. The groups and examples are monoterpenes (C_10_, menthol), sesquiterpenes (C_15_, artemisinin), diterpenes (C_20_, taxol (paclitaxel)), triterpenes (C_30_, saponins), and tetraterpenes (C_40_, carotenoids). **3**—Phenolics, which have aromatic ring compounds with one or more hydroxyl groups. This group is divided into different subgroups: simple phenols (e.g., hydroquinone), flavonoids (e.g., flavonols, flavanons, anthocyanidins), tannins (e.g., ellagitannins), lignins and lignans, stilbenes, and coumarins. **4**—Sulfur-containing compounds derived from amino acids containing sulfur, e.g., glucosinolates, glutathione, and phytoalexines (Figure 1).

Medicinal plants produce a vast array of over 100,000 known low-molecular-weight SMs [10] with beneficial therapeutic and pharmacological effects [6]. The biosynthesis, distribution, and accumulation of SMs are highly species-dependent and reflect the unique physiological and biochemical characteristics of each medicinal plant [9]. For example, in dragon’s head (*Lallemantia iberica*), anthocyanins and essential oils are accumulated in the leaves. In Zingiberaceae plants like ginger (*Zingiber officinale*) and turmeric (*Curcuma longa*) concentrate active ingredients—gingerol and curcumin, respectively—in their roots. In contrast, other plants, such as coffee (*Coffea arabica*), concentrate the active compound, caffeine, in the seeds. The biosynthesis of SMs starts through fundamental metabolic pathways, including glycolysis, the malonic acid pathway, the shikimic acid pathway, and the mevalonic acid pathway, before diverging into a variety of compounds.

The diversification in SMs is heavily influenced by cell type, the plant’s developmental stage, and environmental factors (e.g., temperature, humidity, and light intensity) [11,12]. Also, the SMs pathways and their regulation are highly susceptible to environmental variations because different stresses alter the expression of genes involved in their synthesis [9]. Additionally, reductions in plant populations, cultivation difficulties, low concentrations of essential metabolites in some species, and climate change have impacted the production of some SMs in medicinal plants [12,13].

Plant cell and tissue culture (PCTC) has emerged as a commercially viable solution for the pharmaceutical industry, offering a sustainable alternative to extracting these compounds from wild plants. In vitro elicitation is a key strategy that can activate stress-related pathways, alter cellular redox balance, and upregulate key biosynthetic genes involved in SMs production [11]. Among modern elicitation techniques, nano-elicitors have gained prominence as a practical and eco-friendly approach. Their unique physicochemical properties, such as nanoscale size and high reactivity, enable enhanced interaction with plant cellular machinery, thereby stimulating the biosynthesis of high-value SMs [5]. While nanomaterials (NMs), including metal-based nanoparticles (NPs), nanotubes, nanofibers, nanolayers, and nanosheets, have been widely developed for applications, such as food safety and plant growth, they also show great promise in PCTC.

NMs can modulate defense signaling pathways and redirect metabolic flux, leading to a significant increase in the accumulation of pharmaceutically important compounds, such as alkaloids, phenolics, terpenoids, and saponins [5]. However, despite a growing body of research, the precise mechanisms of nanoparticle-mediated elicitation in plant cell cultures remain poorly understood, and their effects are highly variable, depending on particle type, concentration, and exposure duration [5]. The core of this review is that NPs, beyond their role as simple abiotic stressors, can function as precise regulators of metabolic flux and SMs biosynthesis when optimally applied in plant in vitro systems.

This review seeks to evaluate the potential of nano-elicitors as powerful tools for enhancing SMs production and boosting the yield of valuable bioactive compounds in medicinal plants through PCTC. Furthermore, it aims to identify critical research gaps and outline future perspectives for the controlled and sustainable application of nano-elicitors in plant cell culture.

## 2. Working Methodology

This review was conducted through a comprehensive search of scientific literature related to the use of NMs as elicitors in plant cell, tissue, and hairy root culture systems. Scientific databases including Scopus, Web of Science, PubMed, Research Gate, and Google Scholar were searched using keywords including “cell culture,” “callus,” “hairy roots,” “nanomaterials,” elicitors,” and “metabolites.” Keywords were used in combination with Boolean operators to ensure precise and reproducible results. Specifically, we applied AND to combine different keywords, OR to include synonyms, and NOT to exclude irrelevant terms. All retrieved articles were analyzed and categorized by nanoparticle type, plant culture system, exposure concentration, and SMs type. A comparative evaluation across studies was performed to highlight the effective concentrations, response mechanisms, and potential advantages of nanoparticle-based elicitation for enhancing SMs production.

## 3. Plant Cell and Tissue Culture for SMs Production in Medicinal Plants

In vitro culture techniques present a viable alternative strategy for the efficient production of SMs, which, in natural conditions, is highly dependent on environmental fluctuations [14,15]. This approach enables the generation of substantial quantities within a reduced timeframe under strictly controlled, optimized environmental conditions [16,17]. The quality and quantity of SMs produced in vitro depend on various factors, including the composition of the culture media, pH, and culture environment (temperature, light, agitation, and aeration) [11,15]. Based on the nature of the explant and the nutrients provided in the culture medium, different in vitro culture systems can be established. in vitro culture has evolved into an effective tool for producing industrial and pharmaceutical compounds from plant cells under controlled, aseptic conditions [17,18,19,20]. The most relevant techniques for SM production in cell and tissue cultures include cell suspension culture, callus culture, and hairy root culture [17]. The methods based on PCTC have evolved into a scalable approach that provides precise control over SMs biosynthesis [20,21]. Here, we focused on the effects of different NMs on three applicable methods for producing SMs: callus culture, cell suspension culture, and hairy root culture.

### 3.1. Callus Culture

Callus is a tissue formed primarily from an unorganized mass of meristematic or proembryonic cells, typically induced from different parts of the whole plant or from aseptically germinated seeds [20,22]. Callus culture (CC) is an excellent tissue culture model for studying cellular processes in a tightly controlled environment, with rapid growth and high reproducibility. Moreover, CC serves as a precursor for cell suspension systems, which are increasingly used in the scalable production of bioactive compounds [23]. Unique Advantages of callus culture for SMs production could be detailed as controlled homogeneity and scalability within a uniform microenvironment, seasonal and geographical independence, and the possibility of applying different elicitors as a cutting-edge strategy to produce a wide range of SMs compounds. It is a suitable tool for genetic manipulation and somaclonal variation, as well as a conservation-oriented method for sustainable production. Also, callus cultures bypass geographical and climatic constraints, ensuring year-round, stable metabolite production.

### 3.2. Cell Suspension Culture

Cell suspension culture (CSC) is a crucial technique in cell biology where single cells or small aggregates are cultivated in an agitated liquid medium in a controlled environment [21]. This methodology is particularly effective for unattached cells. It relies on continuous agitation via orbital shakers, spinner flasks, or stirred bioreactors to maintain cells in suspension [24]. The versatility of CSC makes it ideal for high-yield biomanufacturing and provides uniform cell populations [18].

Unique advantages of the CSC method for SMs production include a homogeneous, controlled growth environment over environmental parameters such as pH, temperature, illumination, elicitation protocols, precursor feeding, and bioreactor optimization; better nutrient and oxygen transfer; and scalability in bioreactors [18,20,25]. Plant CSCs are now being employed for in vitro germplasm preservation, recombinant protein expression, scaled-up using bioreactors, transgenic plant development, generation of novel mutants through treating the cells with mutagens, and biotransformation [24]. Cell suspension cultures are also a good source for the examination of metabolomic and proteomic changes that take place during the production of various bioactive compounds [24]. Although CSCs have been successfully used to produce high levels of SMs, these initiatives face challenges, including poor cell efficiency, slow growth, genetic instability of high-producing cell lines, inadequate regulation of cellular differentiation, high costs, and difficult-to-control contamination [25].

### 3.3. Hairy Root Cultures

Hairy root cultures (HRCs) are generally classified as a type of organ culture, since they maintain the differentiated structure and physiological functions of plant roots under in vitro conditions [16].

This technique involves transforming plant explants with *Rhizobium rhizogenes*, which induces hairy roots (HRs) by inserting transfer DNA (T-DNA) [22]. However, this technique is often considered a bridge between organ and cell culture techniques in plant biotechnology [16]. HRCs systems are hormone-independent, genetically stable, and exhibit rapid growth rates due to their genetic transformation with T-DNA [13], which offers a cost-effective method to enhance the production of a wide variety of SMs [22]. HRs are a dependable source with high growth capacity that, when combined with strategies such as precursor feeding, immobilization, biotransformation, and elicitation, can be effective for SM production [26]. Additionally, HRCs tend to be more complex and costly than CSCs [27]. Although CC, CSC, and HRCs each possess their own advantages and limitations, as previously mentioned, all three approaches remain practically valuable and can be effectively employed depending on the specific objectives of the study. For instance, the growth rate and productivity of CC and CSC may be higher than those of HRCs in some cases; however, this depends strongly on plant species, genotype, and the type of SMs [16,18].

## 4. Mechanisms of NPs’ Action on SMs Production

**Figure 2:**
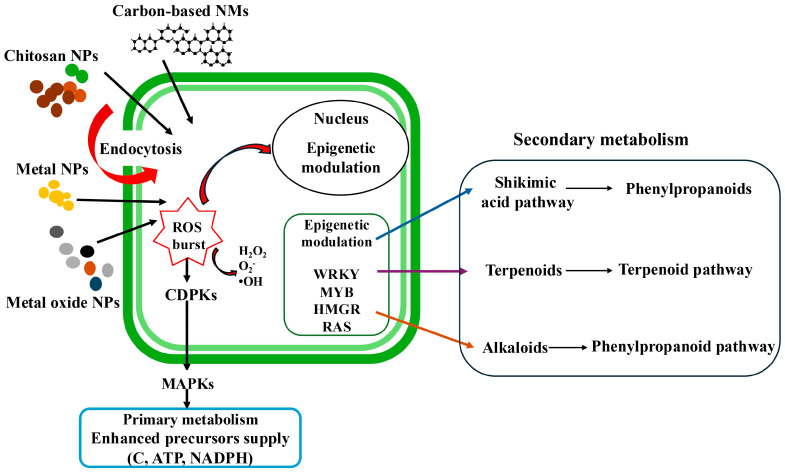
Diagrammatic illustration describing possible mechanisms underlying nanoparticle-mediated elicitation of secondary metabolites in plant cell, callus, and hairy roots cultures.

Nanoparticles have transformed plant biotechnology by providing precise tools for modulating growth, defense, and metabolism. Their functional effects are derived from the physicochemical properties, such as size, shape, surface charge, and composition, which determine the uptake routes, cellular distribution, and biochemical reactivity [28,29]. When properly applied, NPs can amplify the biosynthesis of SMs, strengthen antioxidant systems, and enhance stress tolerance in medicinal plants. Mechanistically, these effects are orchestrated through finely tuned bursts of reactive oxygen species (ROS), calcium signaling, and activation of mitogen-activated protein kinase (MAPK) cascades that converge on the transcriptional reprogramming of biosynthetic genes [30]. The small size, high surface area, surface charge, and capacity for apoplastic or symplastic transport allow NPs to exhibit increased electrostatic interactions with cell membranes. These interactions can subsequently activate and regulate biosynthetic pathways, thereby enhancing SM synthesis in medicinal plant cells [31]. The production of SMs is initiated when nano-elicitors interact with specific receptors located on the plant cell’s plasma membrane (Figure 2). Following this interaction, NPs penetrate the membrane, leading to the generation of ROS, including hydrogen peroxide (H_2_O_2_) and superoxide (O_2_^−^). This ROS burst induces a calcium ion (Ca^2+^) influx into the cytosol through ion channels. This influx acidifies the cytosol, while a simultaneous efflux of chloride (Cl^−^) and potassium (K^+^) ions alkalinizes the extracellular environment. The rise in cytosolic Ca^2+^ is a pivotal elicitation event, as it regulates numerous cellular and physiological processes. Increased cytoplasmic Ca^2^ levels indirectly trigger defense responses via sensors such as Ca^2^-dependent protein kinases (CDPKs). This leads to the reversible phosphorylation and dephosphorylation of cytosolic and plasma membrane proteins, and the subsequent activation of MAPKs and other protein kinases (PKs). Furthermore, elevated intracellular Ca^2+^ enhances ROS production and activates NADPH oxidase. The resulting ROS act as secondary messengers, activating key signaling pathways involving jasmonate, ethylene, and salicylic acid. This cascade ultimately induces the expression of both the defense-related genes and key biosynthetic genes, such as PAL (phenylalanine ammonia-lyase), culminating in the biosynthesis of SMs [28]. It is important to note that the efficiency of SMs generation is highly dependent on several factors, including the plant species, types and concentrations of plant phytohormones, nutritional composition of the medium, the duration of elicitor exposure, and the physical characteristics and concentration of the elicitor itself [14,32].

Beyond these signaling cascades, nanoparticles can also trigger epigenetic modulation, which plays a role in the prolonged activation of secondary metabolism [33]. After NPs uptake and ROS signaling, certain factors and regulators in the nucleus change. These changes include how easily DNA can be accessed or how proteins called histones are modified. These adjustments help control gene activity. Key regulators affected by this process include WRKY transcription factors (named after their conserved WRKYGQK amino acid motif) and MYB transcription factors (originally identified from the myeloblastosis oncogene and which in plants regulate phenylpropanoid metabolism, flavonoid biosynthesis, and multiple stress-responsive pathways) [34,35]; HMGR (3-hydroxy-3-methylglutaryl-CoA reductase), a rate-limiting enzyme in the mevalonate pathway associated with terpenoid biosynthesis [36]; and RAS genes (rolB/rolC-associated signaling), which are linked to enhanced production of specialized metabolites [14]. Nano-elicitors help plant cells produce more phenylpropanoids, terpenoids, and alkaloids by altering signaling pathways and regulating gene expression.

## 5. Types of Nanoparticles as Elicitors in PCTC: Benefits, Mechanisms, and Applications

**Figure 3:**
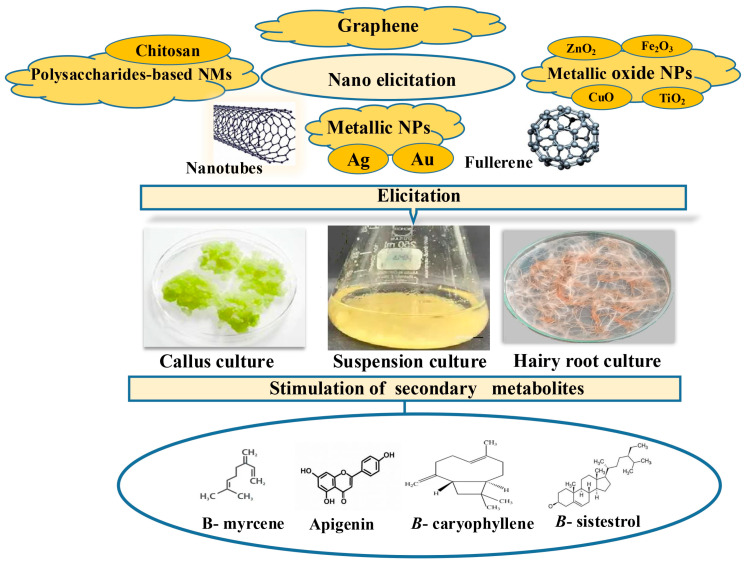
Application of different elicitors on in vitro callus, cell suspension, and hairy roots culture towards the enhancement of secondary metabolites.

According to the literature, different kinds of NPs and NMs are gaining attention for their role as elicitors in plant biotechnology, especially in enhancing the production of valuable SMs in PCTC [37,38]. In this review, we focused on the eliciting effects of different nanoelicitation on SMs stimulation under CC, CSC, and HRCs (Figure 3). The following subsections examine the main classes of NPs, highlighting their biochemical functions, regulatory mechanisms, and their roles as elicitors under operational conditions.

Table 1 summarizes the effects of various NPs used as elicitors across three in vitro culture systems: callus culture, cell suspension culture, and hairy root culture. For each nanoparticle type, the table reports biological responses, including changes in biomass, induction of different SMs, and biochemical alterations. Overall, Table 1 provides a comprehensive overview of nanoparticle-mediated elicitation across diverse medicinal plant culture systems and serves as a helpful basis for selecting suitable elicitors and culture platforms to optimize the production of valuable plant-derived compounds.

### 5.1. Metallic Nanoparticles (MNPs)

Metallic NPs have attracted particular attention as nano-elicitors due to their strong surface reactivity and controlled ion release, which create localized redox signals that reconfigure cellular metabolism. MNPs typically induce a transient ROS burst, upregulate antioxidant enzymes, such as superoxide dismutase and peroxidase, and trigger activation of the phenylpropanoid pathway via PAL [95]. These biochemical events, coupled with hormonal crosstalk, promote both protective and biosynthetic processes while sustaining growth if exposure remains within the hormetic range. In the following subsections, the specific metallic NPs employed in plant cell and tissue culture elicitation are detailed individually, highlighting their mechanisms of action, metabolite-specific responses, and species-dependent differences, thereby providing a comprehensive and nuanced understanding of their elicitation potential.

#### 5.1.1. Silver Nanoparticles

Over the last decade, silver NPs (AgNPs) have emerged as potent elicitors in PCTC, enhancing the biosynthesis of diverse SMs by moderating oxidative effects, defense-related enzymes, and transcription factors, increasing PAL activity and phenylpropanoid flux [46,93,94,96]. In transgenic and in vitro systems, AgNPs also promote alkaloid biosynthesis by priming secondary metabolic pathways through redox-sensitive gene expression networks [45,49]. Ultimately, the balance between ROS generation and detoxification determines whether AgNPs act as stimulators or stressors, underscoring their role as efficient abiotic elicitors that enhance phenolics and alkaloids biosynthesis via redox-regulated, defense-associated pathways. For instance, the stimulatory effects of AgNPs have been reported to increase aloin in *Aloe vera* (L.) [97], hydroxybenzoic and flavanol acids in Bitter guard [40], hyoscine, scopolamine, and hyoscyamine in *Hyoscyamus muticus* [45]. In *Stevia rebaudiana*, two different concentrations of Ag NPs (45 and 60 mg L^−1^) increased the stevioside content of calli, i.e., 1.10 and 1.30-fold higher than the non-elicited treatments [43].

Notably, research indicates that Ag NPs function as effective signal transducers, eliciting an inductive response in Stevia cells. This suggests that Ag NPs could play a significant regulatory role in the glycosyltransferase pathway, thereby enhancing the biosynthesis of stevioside [98]. The concentration of 8 mg L^−1^ of Ag NPs resulted in the highest contents of carvacrol (1.06 mg L^−1^) and thymol (19.75 mg L^−1^) in *Thymus kotschyanus* and *Thymus daenensis*, under callus culture [42]. Elicitation by Ag NPs (10 mg L^−1^) in *Corylus avellana* resulted in the highest paclitaxel production through CSCs [44]. A 24 h post-elicitation period is optimal for maximizing the yield of various polyphenols, including rosmarinic acid, cinnamic acid, and rutin, in Hazel cells [39]. Treatment of *Corylus avellana* (L.) CSCs treated with 5 mg L-1 Ag NPs increased Taxol production [39]. In *Echinacea purpurea*, AgNPs (2-4 mg L^−1^) increased chicoric acid, chlorogenic acid, and caffeic acid levels, particularly in root-derived callus [49]. The treatment by AgNO_3_ elicitation, producing up to 9.5 mg g^−1^ DW chicoric acid within two days. In Perilla frutescens CSC, 100 mg L^−1^ AgNPs elevated caffeic acid (0.57 mg g^−1^ DW) and rutin (1.13 mg g^−1^ DW), coupled with high antioxidant activity and increased PAL, SOD (superoxide dismutase), and POD (peroxidase), indicating preferential activation of the phenylpropanoid branch via ROS-mediated signaling [91]. Similarly, in *Hyoscyamus muticus* HRs transformed with *Agrobacterium rhizogenes* (strain A4), AgNPs (100 mg L^−1^) markedly enhanced the accumulation of tropane alkaloids such as hyoscyamine and scopolamine [45]. Mechanistically, AgNPs operate through transient oxidative signaling that activates antioxidant and biosynthetic enzymes without inducing cytotoxicity.

#### 5.1.2. Gold Nanoparticles

Gold NPs (AuNPs) represent a more recent and equally compelling class of elicitors [99]. They activate auxin-responsive transcription factors, such as ARF1 and ARF3, while upregulating 3-hydroxy-3-methylglutaryl-CoA reductase, the rate-limiting enzyme of the mevalonate pathway, demonstrating how nanogold bridges hormonal regulation with terpenoid biosynthesis [99]. Their actions illustrate how subtle redox cues and hormonal rebalancing can cooperatively drive secondary metabolism when the nanoparticle dosage is carefully optimized. AuNPs have also demonstrated strong elicitation potential in PCTC. Other metallic NPs, such as Fe and Zn, have been used as elicitors in PCTC. Overall, the collective evidence positions Fe and Zn nanostructures as highly promising, low-toxicity alternatives to noble-metal NPs (Ag and Au) in PCTC elicitation.

Different concentrations of FeNPs and ZnNPs (×, 2×, and 4× concentrations of B5 base medium) were used as elicitors on HRCs of fenugreek [54]. The results indicated that using Zn NPs can lead to higher levels of trigonelline (1.85 mg g^−1^ DW) under Zn (2×) treatment compared to the control group. The eliciting effects of other MNPs, such as Co [53] and Pd [55], have been reported (Table 1). The potential eliciting roles of other metallic or metal-based composites (e.g., Al and Co) remain poorly explored in PCTC. The eliciting effects of rare earth and lanthanide metallic NPs, such as cerium (Ce), lanthanum (La), neodymium (Nd), bimetallic and alloy nanostructures (e.g., Ag-Cu, Au-Ag, Co-Ni, among others), and metal–organic/biohybrid metallic composites, e.g., CuHARS (copper high-aspect ratio structures), have not been systematically evaluated.

#### 5.1.3. Selenium-Based Nanoparticles

Selenium NPs (SeNPs) add a unique redox dimension to plant elicitation studies. Acting as both antioxidants and redox regulators, they improve biomass accumulation, enzymatic antioxidant defenses, and biosynthesis of multiple metabolite classes [100]. Their interaction with light signals further magnifies their effects. For instance, under blue LED illumination, SeNPs enhance PAL activity and phenolic biosynthesis in *Santalum album*, whereas selenium-doped ceria–magnetite composites double the chlorogenic acid content by transcriptionally activating key genes [101]. Thus, the efficiency of SeNPs is contingent not only on nanoparticle chemistry but also on the interplay between light quality and metabolic signaling.

### 5.2. Metal Oxide Nanoparticles

In contrast to metallic NPs, metal oxides offer greater chemical stability and lower toxicity. Their capacity to serve as both elicitors and micronutrient sources has positioned them as sustainable alternatives for enhancing SMs production. Metal oxide NPs often act through controlled oxidative priming, elevating antioxidant enzyme activity and triggering metabolic pathways without severely disrupting homeostasis [102].

#### 5.2.1. Iron Oxide NPs

Iron oxide NPs (Fe_3_O_4_ and Fe_2_O_3_) have been used to enhance various SMs in cell cultures [56,57,61]. Their ability to induce significant metabolite gains makes them attractive for scalable biotechnological applications [56]. The significant potential of FeO NPs in photocatalysis arises from their unique magnetic properties and their ability to generate ROS upon light exposure [61,103,104]. According to the literature, iron oxide NPs upregulate PAL and rosmarinic acid synthase (RAS) genes, leading to markedly increased rosmarinic acid accumulation in *Dracocephalum kotschy* HRCs [56]. In addition, FeNPs stimulate secondary metabolism in other species, such as *Hyoscyamus reticulatus*, where they significantly enhance the production of tropane alkaloids, including hyoscyamine and scopolamine [60], through increased antioxidant enzyme activity and metabolic flux. Variations in light exposure can fine-tune these responses, revealing that iron NPs integrate redox control with photoresponsive regulation of cellular functions. In *Artemisia scoparia*, FeONPs (10–15 mg L^−1^) under variable light conditions promoted callus induction (92%), biomass accumulation (33 g L^−1^), and elevated total phenolics (37 mg GAE g^−1^ DW) and antioxidant activity (91%). These findings highlight Fe-based NPs as integrative elicitors that coordinate redox signaling, enzyme activation, and micronutrient balance to enhance metabolic flux toward antioxidant and bioactive compound biosynthesis. Such is the case of γ-Fe_2_O_3_ NPs, whose application in *Bergenia ciliata* CC enhanced biomass, phenolic and flavonoid contents, and antioxidant activity, accompanied by higher enzyme activity and volatile compound production [57]. This behavior suggests that Fe_2_O_3_ provides redox-based cues similar to those of Fe_3_O_4_, but, when combined with hormonal elicitors, it allows the jasmonate signaling pathway to converge with nanoparticle-derived oxidative signals, jointly boosting phenylpropanoid metabolism [66]. Mechanistically, FeONPs induced mild oxidative signaling, modulating SOD, POD, CAT, and APX activities, consistent with redox-mediated stimulation of secondary metabolism, which GC–MS profiling confirmed the presence of bioactive anti-leishmanial compounds under elicitation in *Artemisia scoparia* [61]. In another study, the xanthomicrol, cirsimaritin (as anticancer flavonoids), and isokaempferide content were increased by 11.87, 3.85, and 2.27-fold, respectively, under FeO NPS elicitation in *Dracocephalum kotschyi* HRCs [56].

#### 5.2.2. Zinc Oxide NPs

Zinc is an essential micronutrient that functions as a catalytic cofactor for numerous plant enzymes, reinforcing biochemical activation cascades [105]. Among MONPs, zinc oxide NPs (ZnO NPs) have become one of the most extensively studied nano-elicitors due to their excellent biocompatibility, strong environmental sustainability potential, and demonstrated antioxidant, antifungal, and anticancer properties [105,106]. When applied at appropriate concentrations, engineered ZnO NPs stimulate a wide range of physiological and metabolic responses in plant cells. For example, in *Stevia rebaudiana*, exposure to 1 mg L^−1^ ZnO NPs doubled steviol glycoside content relative to the control and enhanced antioxidant activity, phenolics, and flavonoids. These responses were attributed to moderate ZnO NP–induced oxidative pressure and ROS generation, which activate antioxidant defenses and secondary metabolism, whereas higher concentrations caused metabolic suppression and phytotoxicity, underscoring a dose-dependent balance between stimulation and stress [107]. Optimal outcomes typically occur at mid-range concentrations and short elicitation periods, whereas overdosing reverses the stimulatory effect [107].

Consistent with this hormetic behavior, ZnO NPs have been shown to enhance diverse secondary metabolites (SMs) across multiple in vitro platforms, including callus cultures (CC), cell suspension cultures (CSC), and hairy root cultures (HRCs). In *Linum usitatissimum* CC and CSC, ZnO NP elicitation significantly increased phenolics and lignans [69,70]. In *Thymus kotschyanus*, the highest thymol content (22.83 mg L^−1^) occurred at 150 mg L^−1^ ZnO NPs, while in *T. daenensis* CC, the same concentration yielded maximal carvacrol levels (0.68 mg L^−1^) [108]. In *Silybum marianum* CC, 0.15 mg L^−1^ ZnO NPs produced the highest TPC (37 mg g^−1^ DW), TFC (8.9 mg g^−1^ DW), and silymarin (14.6 mg g^−1^ DW) [72]. Similarly, ZnO NP treatment of *Delonix elata* CC revealed peak phenolic (358.85 µg g^−1^ DW) and flavonoid (112.88 µg g^−1^ DW) accumulation at 30 mg L^−1^, accompanied by significant increases in gallic acid, quercetin, hesperidin, and rutin [109]. In *Hyoscyamus reticulatus* HRCs, ZnO NP elicitation (50–200 mg L^−1^) increased TPC by 3.2-fold and enhanced tropane alkaloids, scopolamine and hyoscyamine, by 1.2-fold [68]. Collectively, these findings demonstrate that ZnO NPs act as versatile and potent elicitors across plant tissue culture systems, with their efficacy strongly governed by concentration, exposure time, and species-specific metabolic capacity.

#### 5.2.3. Copper Oxide NPs

Copper (Cu) is an essential micronutrient for plants and plays a critical role in regulating both primary and SMs in medicinal species. A growing body of research has explored the effects of copper oxide NPs (CuO NPs) on plant systems, highlighting their potential to influence biochemical pathways and SMs production [64]. CuO NPs combine trace-nutrient functionality with potent redox activity, resulting in notable increases in both phenolics and alkaloids [110]. CuO NPs activate WRKY, leading to the upregulation of pathway genes, such as tyrosine decarboxylase and several O- and N-demethylases in the alkaloid biosynthetic chain [70]. Various studies have demonstrated the effectiveness of CuO NPs under PCTC.

In *Gymnema sylvestris*, various concentrations of CuO NPs (1, 3, and 5 mg L^−1^) effectively increased gymnemic acid production by CSCs [64], resulting in a 2.3-fold increase compared to the control treatment. Callus elicitation of *Ocimum basilicum* showed that Murashige and Skoog media supplemented with 10  mg L^−1^ CuO NPs resulted in the highest contents for phenolic (27.5 mg g^−1^ DW), flavonoids (9.1 mg g^−1^ DW), rosmarinic acid (11.4 mg g^−1^ DW), chicoric acid (16.6 mg g^−1^ DW), and eugenol (0.21 mg g^−1^ DW) [74]. In *Papaver orientale*, a species valued for its benzylisoquinoline alkaloids, both chemically synthesized and green-derived CuO NPs (20–40 mg L^−1^) increased H_2_O_2_ accumulation and antioxidant enzyme activity, establishing oxidative stress as the initiating signal at 20 mg L^−1^ for 48 h [65]. The expression of key genes in the benzylisoquinoline alkaloid (BIA) pathway (PsWRKY, TYDC, SalSyn, SalAT, T6ODM, CODM) was upregulated, leading to higher levels of thebaine, codeine, and morphine [65]. This consistency between oxidative markers, transcriptional activation, and metabolite accumulation supports a ROS-to-transcription cascade as the central mechanism of CuO NP elicitation. Optimal elicitation typically occurs at moderate doses and longer exposure times, where oxidative signaling remains stimulatory rather than detrimental [111,112].

#### 5.2.4. Cerium Oxide NPs

Cerium oxide NPs (CeO_2_ NPs) occupy a special niche among elicitors because of their reversible redox states (Ce^3+^/Ce^4+^) to enable ROS buffering and redox homeostasis [113]. When applied singly or in combination with iron oxides and selenium dopants, they markedly enhanced chlorogenic acid biosynthesis and broadened the phenolic profiles [114]. These effects extend beyond stress induction involving the transcriptional reprogramming of biosynthetic genes and metabolic intermediates; this performance illustrates a sophisticated biochemical leverage achievable through composite nanoformulations.

#### 5.2.5. Silicon-Based NPs

Silicon dioxide NPs (SiO_2_ NPs) significantly modulate physiological mechanisms that enhance plant resilience against environmental stressors [115]. This efficacy is primarily attributed to the well-documented beneficial role of elemental silicon (Si) in promoting overall plant development and growth [115]. Nano elicitation with SiO_2_ NPs has increased the TPC and TFC in *Ammi visnaga* (15 mg L^−1^) [84] and *Hyoscyamus reticulatus* (100 and 200 mg L^−1^) [82]. Treatment of *H. reticulatus* HRs with 100 mg L^−1^ SiO_2_ NPs for 24 h resulted in the highest accumulation of the tropane alkaloids hyoscyamine and scopolamine, reaching levels of 140.15 μg/g FW and 67.71 μg g^−1^ FW, respectively [82]. This represented a dramatic increase of 1212% in hyoscyamine and a 272% increase in scopolamine production compared to the untreated control cultures. Other important MO NPs, such as TiO_2_ NPs, have been identified as significant elicitors [31,79] that promote key developmental processes in plants, such as cell division, cell expansion, and callus formation [78]. Furthermore, their physiological role extends to mimicking plant growth hormones [80]. The elicitation effects of TiO2 NPs on HRs of *Saponaria officinalis* L. showed that the highest rate of TPC (9.79 mg GAE g^−1^ FW) and total flavonoids content (TFC) (1.06 mg QE g^−1^ FW) were obtained in the treated HRs with 50 and 30 mg L^−1^ of TiO_2_ NPs under 24 and 48 h of treatments, respectively [78]. According to the literature, the eliciting effects of ZrO_2_ (zirconia), MgO, CaO, MnO, CeO_2_, NiO, CoO, and Cr_2_O_3_ oxides have not been studied under PCTC. These MO NPs lack mechanistic insights and a deficiency in dose-dependent toxicity. Most of the experiments have been limited to small-scale, so the industrial scalability of oxide-based elicitation remains untested.

### 5.3. Carbon-Based NMs

Carbon-based nanomaterials, including carbon nanotubes, graphene derivatives, and fullerenes, exert multifunctional effects by interacting with membranes and influencing electrical potential. This interaction reconfigures cellular redox balance and gene expression, enhancing phenylpropanoid and alkaloid pathways [113,116]. So, recent research suggests that carbon NMs may function as abiotic elicitors, enhancing the biosynthesis of SMs through various biochemical pathways [117]. The elicitation response observed in plant cell cultures is highly dependent on the physicochemical properties of these NMs, including their specific type, surface reactivity, functional groups, and tendency to agglomerate [86]. Hence, carbon nanotubes (CNTs) may be a suitable candidate for use as highly potent elicitors in PCTC, thereby modifying plant growth [38].

#### 5.3.1. Multiwalled Carbon Nanotubes

Functionalized multiwalled carbon nanotubes (MWCNTs) have demonstrated pronounced elicitation effects even at low-to-moderate doses [118]. They promote morphogenesis and activate genes linked to alkaloid and phenylpropanoid biosynthesis (e.g., tyrosine aminotransferase, ornithine decarboxylase, putrescine N-methyltransferase) [119,120]. However, their hormetic nature is evident; high concentrations often result in cytotoxicity and excessive DNA methylation, emphasizing the narrow operational window for beneficial outcomes [121].

The stimulatory effects of CNTs [85,88] and MWCNTs [38] have been reported on the enhancement of SMs (Table 1). However, other researchers reported that MWCNTs at 5–500 µg mL^−1^ [37] enhanced cell growth compared to the control in tobacco callus culture. Thymoquinone (TQ), as the main active ingredient of the SMs, has been considered as an anti-liver cancer agent alone or in combination with other drugs [63]. According to the findings of [63], the highest value for TQ (295 mg L^−1^) was observed under treatment with FeO_3_-CTs NPs (100 mg L^−1^).

#### 5.3.2. Graphene

Graphene oxide (GO) and reduced graphene oxide (rGO) have emerged as potent nano-elicitors for enhancing SM biosynthesis through stimulating antioxidant systems [122]. These NMs function as effective elicitors in plant cell cultures owing to their unique surface properties, such as high surface area, reactive functional groups, and tunable charge, as well as their strong interaction with cellular pathways. These interactions can enhance water transport, active stress-response signaling, and stimulate the biosynthesis os specialized metabolites, ultimately promoting regeneration and biomass accumulation [89]. The positive effects of GO NPs on the enhancement of TPC and TFC have been reported in *P. major* [86] and *Lepidium sativum* L. [87]. A comparison between rGO and CNTs showed that the callus treated with reduced GO showed increased TPC (107.4 µg GAE mL^−1^) and TFC (8.06 µg QE mL^−1^) as compared to TPC (32.3 µg GAE mL^−1^) and TFC (7.11 µg QE mL^−1^) observed in callus treated with CNTs. A recent study demonstrated that carbon-based NMs, particularly rGO, effectively promoted regeneration and SMs accumulation in *Fagonia indica* callus culture [89]. At low concentrations (1–2 mg L^−1^), rGO induced both shoot and root formation and achieved the highest biomass, TPC (107.4 µg GAE mL^−1^), and TFC (8.06 µg QE mL^−1^), outperforming CNTs. HPLC analyses confirmed enhanced biosynthesis of quercetin and gallic acid, indicating that rGO acts as a redox-modulating, biocompatible elicitor that reprograms secondary metabolism. The use of functionalized carbon-based NMs (e.g., carboxylated carbon nanotubes, aminated graphene) may enhance cellular uptake and signaling and, through PCTC, increase SMs. Investigating synergistic combinations of carbon-based NMs with metallic NPs or classical elicitors is needed. To the best of the authors’ knowledge, there are no studies on the elicitation effects of fullerenes (C60) and carbon quantum dots as elicitors in PCTC.

### 5.4. Polysaccharide-Based NMs

Polysaccharide-derived NMs, notably those based on chitosan and chitin, provide biocompatible and biodegradable elicitation pathways [123]. Unlike metallic NPs, plant cells perceive these NMs as pathogen or damage-associated molecular patterns, leading to innate immune activation and metabolic enhancement. Their structural similarity to natural polymers allows them to activate SMs biosynthesis with minimal cytotoxicity, positioning them as sustainable elicitors in plant biotechnological applications [124].

#### 5.4.1. Chitosan

Chitosan NPs have unique properties, such as being nontoxic, biocompatible, and biodegradable, and exhibit stimulatory and antibacterial effects, which have been used for SMs production [90].

Chitosan NPs bind to negatively charged cell wall sites, initiating defense signaling and PAL activation [90,125]. When used as carriers for signaling molecules, such as methyl jasmonate, they enable controlled release, extending the duration of metabolic activation [92,126]. This dual functionality, both as an elicitor and a carrier, illustrates how biodegradable nanopolymers can enhance metabolite biosynthesis while ensuring environmental and biological compatibility.

The mixture of chitosan NPs and methyl jasmonate improved the production of phenols and flavonoids in SCS [127]. Under chitosan NPs elicitation in *Silybium marianum*, the highest record of taxifolin, silychristin, silybin B, and isosilybin A was obtained under 50 mg L^−1^ chitosan NPs that were coated with 40 mg L^−1^ phenylalanine [90]. The positive effect of chitosan NPs on artemisinin production in *Artemisia annua* CSC has also been reported [53]. Betulin and betulinic acid are two triterpenes with diverse pharmacological and physiological actions. An elicitation treatment with 0.5% cellulose nanofiber resulted in the highest betulin accumulation, yielding 0.7 mg g^−1^ in *Betula pendula* [91].

#### 5.4.2. Chitin

Nanochitin operates through a closely related yet distinct mechanism, directly engaging plant immune receptors to induce robust defense metabolism by activating MAPK signaling cascades and subsequent triterpenoid pathways [91,128]. Short exposures to low concentrations favor both biomass and secondary metabolism, whereas prolonged treatment may shift the balance toward defense at the expense of growth. Compared to chitosan, nanochitin is a more potent activator of immune and phenolic pathways, making it particularly suitable for stimulating the production of high-value metabolites in controlled cultures [129]. According to the literature, no study has been done on the eliciting effect of chitin in PCTC.

### 5.5. Quantum Dots

Quantum dots (QDs) represent a new generation of elicitors effective at trace concentrations due to tunable redox properties [130,131]. Unlike polysaccharide or metallic NPs, they do not mimic biological ligands operating through redox-mediated signal transduction [132]. Carbon dots (CDs) are an example of this mode of action. For instance, when applied to *Lactica sativa* L. at low doses, they simultaneously promote biomass accumulation and redirect carbon flux toward triterpenoid and sterol biosynthesis [133]. Their elicitation mechanism involves the generation of superoxide radicals, which trigger antioxidant enzyme cascades and downstream Ca^2+^ and MAPK signaling [134]. Advanced modeling using artificial neural networks has captured these complex responses with high predictive accuracy, providing a robust computational framework for optimizing elicitor dose and timing to maximize productivity. Graphene-based quantum nanocomposites, such as rGO/Fe_3_O_4_ hybrids, illustrate how material engineering can balance elicitation intensity with cellular tolerance [135]. These systems enhance triterpenoid production while maintaining physiological stability, as their moderated redox activity induces metabolic shifts without causing excessive stress [136]. Such composites represent a promising path toward precision-controlled elicitation with a minimal ecological footprint.

### 5.6. Composite/ Hybrid NMs

Few studies have shown the effects of hybrid nanomaterials on SMs through PCTC [52]. In CC of *Nigella sativa* L., the highest levels of TPC, TFC, and amino acids were observed under Fe chitosan-coated nano iron (FeO_3_- CTs) (100 mg L^−1^) [63]. In comparison, FeO_3_- CTs at 100 and 200 mg L^−1^ exhibited significant effects on TPC, TFC, quercetin, gallic acid, and thymoquinone [63]. Under CC of *Ammi visnaga* L., a comparison between SiO_2_ NPs and GO-coated SiO_2_ NPs showed a superiority of GO-SiO_2_ NPs in stimulating TPC and TFC in comparison to SiO_2_ NPs [84]. Hybrid composites (GO–SiO_2_ and rGO/Fe_3_O_4_) stabilize redox states, preventing overoxidation and ensuring balanced metabolic activation [87,136]. These materials thus act as redox “priming agents,” improving plant readiness for stress and boosting secondary metabolite production through subtle oxidative and transcriptional cues under PCTC [137].

### 5.7. Non-Metallic Elements

According to the literature, few studies have examined the effects of non-metallic element NPs. HPLC quantitative analysis demonstrated a significant increase in phenolic and flavonoid compounds in *Lotus arabicus* CC treated with sulfur NPs (S NPs), particularly at 100 mg L^−1^, resulting in a 1.1-fold increase compared to the control [93]. The data further indicated a substantial increase in specific benzoic acid derivatives under the 100 mg L^−1^ S NP treatment. Notably, gallic acid increased by 1.37-fold, methyl gallate by 22.9-fold, and syringic acid by 2.4-fold. In contrast, the highest concentrations of ellagic acid and vanillin were observed at lower SNP doses of 25 mg L^−1^ and 50 mg L^−1^, respectively. To the authors’ knowledge, there are no studies involving nano-nitrogen compounds, selenium NPs, boron-based NPs, phosphorus-based NMs, or halogen-based NMs (Cl, Br, I) as elicitors in in vitro systems.

### 5.8. Integrative Considerations for Design and Scale-Up

Across NPs classes, a unifying principle emerges: elicitation efficacy follows dose–time hormesis, where short pulses at low-to-moderate concentrations maximize SMs while avoiding oxidative damage [138]. Surface functionalization, dopant chemistry, and composite formation often determine whether a nanoparticle acts as a stimulant or stressor [139]. Factors such as auxin balance, cell culture age, and light quality are vital for regulating nanoparticle signaling in both environmental and cell culture contexts [131]. To confirm mechanisms, it is crucial to combine ROS kinetics, antioxidant enzyme assays, and transcriptional profiling to establish causal links between nanoparticle properties and their metabolic impacts. For scaling up, emphasis should be placed on biocompatible formulations like ZnO, iron oxides, and polysaccharide carriers, as well as systems that are either magnetically recoverable or biodegradable [140]. By integrating experimental optimization with predictive modeling, it will be feasible to develop nano-elicitation strategies that enhance metabolite production in an efficient, sustainable, and reliable manner.

## 6. Advantages and Challenges

### 6.1. Advantages

As previously mentioned, PCTC supports agricultural activities by addressing issues such as mass propagation, germplasm conservation, genetic engineering, aseptic plant production, and increased SM output [117,141,142]. First, it was reported that NPs serve as efficient sterilizing agents at low concentrations, reducing microbial contamination during explant preparation and enhancing the overall success of the in vitro culture process [143]. Silver NPs are among the most widely used NPs in the disinfection of explants and culture media [31,144]. NPs, such as CuO, ZnO, TiO_2_, and graphite, have demonstrated decontamination power; however, there are no ionic counterparts to compare their effectiveness. Considering the eliciting effects of NMs under PCTC, the spectrum of used NPs as elicitors is significantly higher compared to traditional elicitors (such as ethylene, abscisic acid, gibberellic acid, and methyl jasmonate) [145]. Moreover, NPs can serve as carriers for genetic material, facilitating gene delivery and transformation processes in plant cells. The application of NPs also reduces the levels of plant phytohormones in culture media and promotes more natural growth patterns. Additionally, NPs can enhance nutrient and water uptake, thereby improving plant vigor and resilience under stress conditions. Overall, the use of NPs in plant tissue culture offers a multifaceted approach to enhancing plant growth, SMs production, and genetic transformation, thereby advancing the field of plant biotechnology. Nanomaterials showed greater stability compared with traditional elicitors. Their unique physicochemical properties, such as a high surface-area-to-volume ratio and the ability to be functionalized, enhance their dispersion and interaction with plant cells. For instance, studies have demonstrated that functionalized cerium and iron oxide NPs maintain colloidal stability in cell culture media, ensuring prolonged and consistent elicitation effects [146]. In contrast, conventional elicitors, including plant hormones and microbial extracts, often face challenges such as degradation, compositional variability, and batch-to-batch inconsistencies, leading to unpredictable responses in plant cultures [147]. The stability of NPs enables more controlled and reproducible elicitation of secondary metabolites, making them valuable tools for enhancing SM production in plant tissue cultures.

### 6.2. Research Gaps and Limitations

Despite the significant advantages shown by nanomaterials as elicitors in PCTC, the application of NPs as elicitors in plant tissue culture systems faces several substantial gaps and challenges that must be addressed to ensure reproducible and safe outcomes.

According to the literature, there is insufficient data available on optimal doses and toxic concentrations (dose-dependent responses) as threshold limits for many new types of NMs used as elicitors under PCTC. The magnitude and direction of metabolic responses to NPs are strongly concentration-and species-dependent. According to the literature, low to moderate NPs concentrations often increased SMs accumulation, whereas higher concentrations may lead to lipid peroxidation, membrane damage, DNA degradation, and inhibition of key antioxidant enzymes, ultimately reducing cell viability and growth under PCTC [6,117,148]. Long-term exposure to NPs also raises concerns about genomic instability, somaclonal variation, and changes in gene expression, which can compromise the regenerative capacity and genetic fidelity of plant cell culture [117,148]. Thus, one of the major concerns is about the potential toxicity of the used NPs, which depends on inappropriate concentrations, particle sizes of NPs, shapes, or chemical compositions of the used material [117,149], the exposure time, and the culture condition [149].

There is a lack of information on the scalability and biosafety of using NMs in large-scale methods, such as HRCs or CSCs. In addition, non-uniform dispersion and aggregation of NPs within the culture medium can decrease their bioavailability and efficacy. Additionally, the interactions with ions, organic compounds, and other components of the medium often lead to agglomeration or surface modifications that limit cellular contact [149]. The efficiency of cellular internalization of NMs depends on different factors such as nanoparticle size, coating, and their stability, which determine whether entry occurs via diffusion, endocytosis, or active transport [150]. According to the literature review, few studies have been reported regarding the mechanisms of NPs uptake into plant cells, translocation, and intracellular signaling under PCTC conditions. Also, the elicitation pathways for non-metallic NMs (ROS modulation, nutrient signaling, and membrane interactions) are poorly understood. Most studies have focused on MNP and MONPs in plant cells and HRCs, and non-metallic elicitors remain unexplored. Also, there is a lack of comparative studies for comparing metallic vs. non-metallic nanomaterials in the same culture system. Also, there is limited information on the combination of current elicitors (e.g., jasmonic acid, salicylic acid, biotic elicitors) with NMs.

Finally, environmental and biosafety implications must be considered, as residual NPs in culture biomass or effluents could accumulate in ecosystems or enter the food chain. Therefore, comprehensive risk assessment and life-cycle evaluation of NPs use in plant biotechnology are essential before large-scale application [147,148].

## 7. Conclusions and Future Recommendations

The present review highlighted the remarkable potential of different nanomaterials as novel and efficient elicitors in plant in vitro culture systems, including callus, suspension, and hairy root cultures. Nanoparticulate materials, including Ag, ZnO, CuO, TiO_2_, Fe_3_O_4_, and carbon-based NMs (graphene, carbon nanotubes), have been reported to modulate plant SMs through their unique physicochemical properties and ultimately enhance the biosynthesis of valuable SMs such as alkaloids, flavonoids, phenolic acids, terpenoids, and saponins. Overall, nanoparticle-mediated elicitation represents a promising, controllable, and sustainable strategy for enhancing the production of bioactive compounds in medicinal plants. However, to optimize nanomaterial application, further studies are required to:

(1) Elucidate the precise molecular mechanisms of nanoparticle–plant interactions, assessing long-term cytotoxic effects.
(2) Establish standard guidelines for safe and efficient use of industrial-scale production of high-value SMs.
(3) Evaluate the synergistic combinations of NPs with biotic elicitors for more potent effects.
(4) Develop smart NPs designed for specific metabolic pathways.
(5) Elucidate nanoparticle–cell interactions, redox regulation, and gene-level responses across different metal-based nano elicitors.
(6) Integrate nanotechnology with omics-based approaches (genomics, transcriptomics, metabolomics) and metabolic engineering to reveal molecular mechanisms.
(7) Include membrane filters with a pore size of 0.1 µm (when possible) for removing bacteria, fungi, and mycoplasma to prevent tissue culture contamination.

## Figures and Tables

**Table 1:** Effects of different nano-elicitation methods on the accumulation of secondary metabolites in various medicinal plants under callus, cell suspension, and hairy roots culture.

Nanomaterial	Type of Culture	Plant Name	Secondary Metabolite	Reference
Metallic NPs				
Ag	CSC	*Aloe vera*	Increase aloin	
Ag	CSC	*Corylus avellana*	Increase taxol and baccatin	[39]
Ag	CSC	Bitter guard	Increase TFC, TPC, hydroxycinnamic, hydroxybenzoic, and flavanol acids	[40]
Ag	CC	*Caralluma tuberculata*	Increase TPC and TFC	[41]
Ag	CC	*T vulgaris*, *T. daenensis*, *T. Kotschyanus*	Increase thymol and carvacrol	[42]
Ag	CC	*Stevia rebaudiana*	Increase steviol glycosides (stevioside and rebaudioside A	[43]
Ag	CC	*Zataria multiflora*	Increase thymol and carvacrol	[42]
Ag	CSC	*Corylus avellana*	Increase paclitaxel, taxol, baccatin	[44]
Ag	HRCs	*Hyoscyamus muticus*	Increase hyoscine, scopolamine, hyoscyamine	[45]
Ag	CC	*Ruta chalepensis*	Increase TPC, tannin, TFC, flavanols	[46]
Au		*Artemisia absinthium*	Increase TPC and TFC	[47]
Au	CSC	*Taxus baccato*	Increase TPC and taxane	[48]
Ag	CC, CSC	*Echinacea purpurea*	Cichoric acid, chlorogenic acid, caffeic acid	[49]
Ag	HRCs	*Hyoscyamus muticus*	Tropane alkaloids (hyoscyamine, scopolamine)	[45]
Ag	CSC	*Perilla frutescens*	Caffeic acid, rutin; enhanced PAL, SOD, POD activity and antioxidant capacity via ROS-mediated phenylpropanoid activation	[50]
Ag and Au	CC	*Prunella vulgaris*	Callus proliferation, increase TPC, TFC	[51]
Ag-SiO_2_	HRCs	*Artemisia annua*	Increase artemisinin	[52]
Co	CSC	*Artemisia annua*	Increase artemisinin	[53]
Zn	HRCs	*Salvia miltiorrhiza* Bunge	Increase tanshinone, rosmarinic acid, caffeic acid, and salvianolic acid	[26]
Zn and Fe	HRCs	*Trigonella foenumgraecum *	Increase TPC, TFC, and trigonelline	[54]
Ag, Au, Cu, Pd	CSC	*Hypericum perforatum*	Increase bisxanthone, gancaonin O, fusaroskyrin hyperxanthone C (Au), apigenin (Cu), emodin (Pd),	[55]
**Metallic oxide NPs**				
FeO	HRCs	*Dracocephalum kotschyi*	Increase TPC, TFC, rosmarinic acid, xanthomicrol, cirsimaritin, and isokaempferide	[56]
FeO	CC	*Bergenia ciliata*	Increase TPC, TFC, and volatile compounds	[57]
FeO and ZnO	CSC	*Hypericum perforatum*	Increase hypericin and hyperforin	[58]
Fe_3_O_4_	CSC	*Dracephalum polychaetum*	Increase naringin, apigenin, rutin, rosmarinic acid, quercetin, thymol, and carvacrol	[59]
Fe_3_O_4_	HRCs	*Hyoscyamus reticulatus*	Increase hyoscyamine and scopolamine	[60]
Fe_3_O_4_	CC and CSC	*Artemisia scoparia*	Increase TPC, TFC, and volatile constituents	[61]
Fe_3_O_4_-β-cyclodextrin	CSC	*Vitis vinifera*	Increase resveratrol	[62]
F_e_O_3_-CTs	CC	*Nigella sativa*	Increase TPC, TFC, thymoquinone	[63]
CuO	CSC	*Gymnema sylvestre*	Increase TPC, TFC, and gymnemic acid II	[64]
CuO	CSC	*Papaver orientale*	Benzylisoquinoline alkaloids (thebaine, codeine, morphine)	[65]
Fe-ZnO	CSC	*Fagonia indica*	Increase TPC and epigallocatechin gallate	[66]
ZnO	CC	*Stevia rebaudiana*	Increase TPC, TFC,	[67]
ZnO	HRCs	*Hyoscyamus reticulatus*	Increase tropane alkaloids	[68]
ZnO	CSC	*Linum usitatissimum *	Increase lignans (secoisolariciresinol diglucoside, lariciresinol diglucoside)	[69]
ZnO	CC	*T. vulgaris*, *T. daenensis*, *T. Kotschyanus*	Increase thymol and carvacrol	[42]
ZnO	CC	*Linum usitatissimum*	Increase TPC, TFC, secoisolariciresinol diglucoside, lariciresinol diglucoside, dehydrodiconiferyl alcohol glucoside (25 mg L^−1^)	[70]
ZnO	CSC	*Nigella sativa*	Increase TPC, TFC, and thymoquinone	[71]
>ZnO	CC	*Silybum marianum *	Increase TPC, TFC, and silymarin	[72]
ZnO, CuO and CoO	CC	*Artemisia annua*	Increase TPC, TFC, rutin, gallic acid, and caffeic acid	[73]
CuO and MnO	CC	*Ocimum basilicum*	Increase TPC, TFC, rosmarinic acid, chicoric acid, eugenol	[74]
CuO and ZnO		*Glycyrrhiza glabra*	Increase glycyrrhizin	[75]
MgO and CuO	CC	*Punica granatum*	Increase TPC, total tannins, gallic acid, ellagic acid, tannic acid	[76]
TiO_2_	CC	*Salvia tebesana*	Increase TPC and TFC	[77]
TiO_2_	HRCs	*Saponaria officinalis*	Increase TPC, total TFC, and SO_6_ anticancer protein	[78]
TiO_2_	CC	*Teucrium polium*	Increase TFC, flavones, rosmarinic acid,	[79]
TiO_2_	HRCs	*Dracocephalum kotschyi*	Increase TPC, TFC, rosmarinic acid, xanthomicrol, cirsimaritin,	[80]
Al_2_O_3_ and WO_3_	CC	*Datura* spp.	Increase TPC, TFC, and alkaloids	[5]
Al_2_O_3_	CSC	Tobacco	Increase total phenolics	[81]
SiO_2_	HRCs	*Hyoscyamus* spp.	Increase TPC, TFC, tropane alkaloids (, scopolamine)	[82]
SiO_2_	CC	*Tagetes erecta*	Increase phenolic compounds	[83]
SiO_2_	CC	*Ammi visnaga*	Increase TPC and TFC	[84]
SiO_2_	CC	*Caralluma tuberculata*	Increase TPC, TFC, coumarins, gallic acid, caffeic acid, ferulic acid, catechin, quercetin, and rutin	[41]
**Carbon–based NMs**				
CNTs	CC	*Satureja khuzestanica *	Increase TPC, TFC, rosmarinic acid, caffeic acid	[85]
CNTs	CC	Tobacco	Cell growth and division	[37]
MWCNTs	CC	*Catharanthus roseus*	Increase TFC	[38]
GO	CC	*P. major*	Increase TPC, TFC	[86]
GO	CC	*Lepidium sativum *	Increase TPC, TFC, and anthocyanin	[87]
CNTs and GO	CC	*Fagonia indica*	Increase TPC, TFC, caffeic acid, rutin, and benzoic acid	[88]
CNTs and reduced GO		*Fagonia indica*	Increase TPC, TFC, quercetin, and gallic acid	[89]
**Polymeric NMs**				
Chitosan	CC	*Silybium marianum*	Increase silymarin isomers (taxifolin and silydianin) and some phenolics (P-OH-benzoic acid and protocatechuic acid	[90]
Cellulose nanofiber, chitosan nanofiber, chitin nanofiber	CC	*Betula pendula*	Increase betulin and betulinic acid	[91]
Chitosan	CSC	*Artemisia annua*	Increase artemisinin	[53]
Methyl jasmonate-loaded chitosan NPs (MJ-CNPs)	CC	*Oriza sativa*	Enhanced production of phenolics and flavonoids; prolonged PAL activity	[92]
Sulfur	CC	*Lotus arabicus*	Increase ellagic acid, vanillin, gallic acid (1.37-fold), methyl gallate (22.9-fold), and syringic acid (2.4-fold) under 100 mg L^−1^	[93]
**Composite/hybrid NMs **				
GO-SiO_2_	CC	*Ammi visnaga*	Increase TPC, TFC	[84]
GO-SiO_2_	CC	*Levisticum officinale* Koch.	Increase TPC, TFC	[94]

CC: Callus culture; CSC: Cell suspension culture; HRCs: Hairy root culture; TPC: Total phenolics content; TFC: Total flavonoids content; PAL: Phenylalanine ammonia-lyase; SOD: Superoxide dismutase; POD: Peroxidase.

## Data Availability

No new data were created or analyzed in this study.
